# A Review of Research Progress on the Microbial or Enzymatic Degradation and Mechanism of Aflatoxin B1

**DOI:** 10.4014/jmb.2504.04044

**Published:** 2025-08-18

**Authors:** Aiyuan Zhang, Jiguo Yang

**Affiliations:** 1 College of Food Science and Engineering, South China University of Technology, Guangzhou, P.R. China; 2 South China Institute of Collaborative Innovation, Dongguan, P.R. China

**Keywords:** Aflatoxins, bacteria, fungi, biodegradation, mechanism

## Abstract

Aflatoxins (AFs) are secondary metabolites produced by toxigenic *Aspergillus* species, which are highly toxic and exhibit carcinogenic, mutagenic, and teratogenic properties, posing a significant threat to human and animal health. Cereals and their products are the primary targets for aflatoxin-producing fungal contamination, causing substantial losses to the livestock and food industries. Due to the high toxicity and widespread contamination of aflatoxin B1 (AFB1), the prevention and degradation of AFs in cereals and their products are essential. This review summarizes the AFB1-degrading bacteria, such as *Bacillus*, *Actinomycetes*, and *γ-Proteobacteria*, as well as fungi, including *Aspergillus* and *Basidiomycota*. It also provides an overview and discussion of the chemical structures and toxicity of the degradation products. Additionally, the review delves into the possible oxidative, reductive, and hydrolytic mechanisms of AFB1 enzymatic degradation. The aim of this study is to provide a reference for the biological degradation of AFB1 and to promote further research in this area.

## Introduction

Mycotoxins are toxic secondary metabolites produced by certain strains of toxigenic fungi from genera such as *Aspergillus*, *Fusarium*, *Alternaria*, and *Penicillium* during their growth [1]. These low molecular weight natural compounds pose significant threat to human and animal health [2], having carcinogenic, mutagenic, teratogenic, and hepatotoxic properties [3]. Cereals are the primary targets for mycotoxin-producing strains [4]. The Food and Agriculture Organization (FAO) estimates that about 25% of the world's cereals are contaminated with mycotoxins each year [5].

**Fig. 1:**
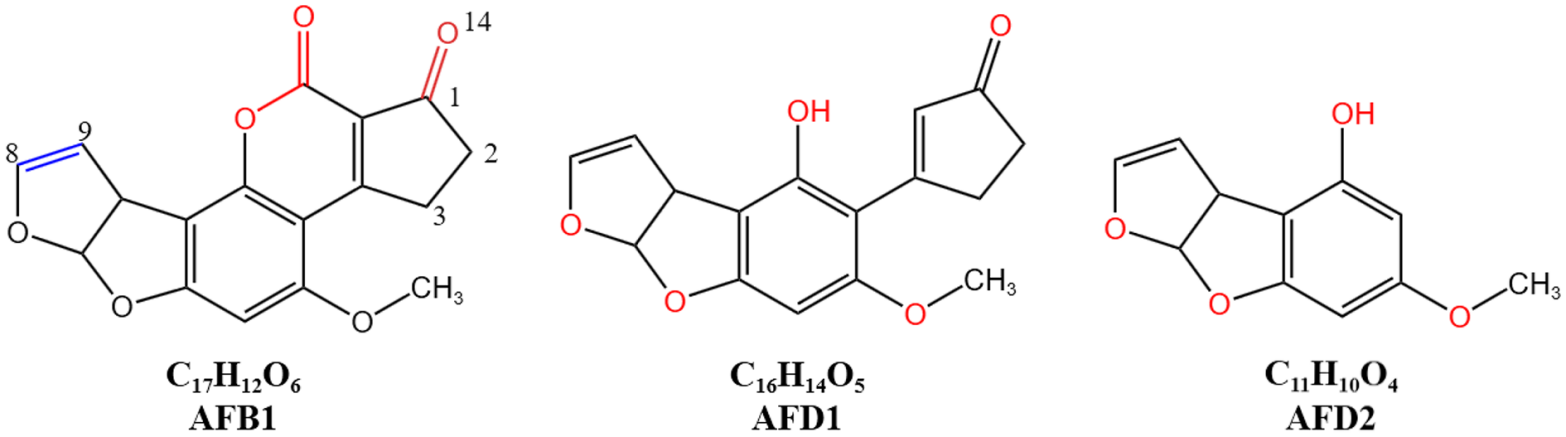
Chemical structure of AFB1, AFD1 and AFD2. The primary toxic site of AFB1 is the double bond between the C8 and C9 atoms within the furan ring, which is indicated in blue font in the molecular structure of AFB1. The second toxic site is the lactone ring within the coumarin nucleus, which is denoted by the red-colored ester bond in the molecular structure of AFB1. The third toxic site is located on the cyclopentenone ring of AFB1, specifically at positions 1, 2, 3, and 14. AFD1 is the product of AFB1 after the elimination of the lactone carbonyl; AFD2 is the product resulting from the cleavage and elimination of the cyclopentenone ring in AFD1.

AFs are mainly produced by molds such as *Aspergillus flavus* and *Aspergillus parasiticus* from the genus *Aspergillus* during their metabolism in foods like peanuts, corn, rice, nuts, cocoa beans, dried fruits, spices, and crude vegetable oils [6]. AFs are derivatives of difuranocoumarin, featuring a dibenzofuran group and a five-membered ketone ring connected to the coumarin nucleus [7], as shown in Fig. 1. The most common aflatoxin is AFB1, which is the most toxic among the 20 types of AFs [8].

The primary toxic site of AFB1 is the double bond between the C8 and C9 atoms in the furan ring. After ingestion, AFB1 is metabolized by cytochrome P_450_ enzymes into a highly reactive electrophilic intermediate, AFB1-exo-8,9-epoxide (AFBO). AFBO can bind to the N_7_ position of guanine in DNA, forming an AFB1-N_7_-guanine adduct, which induces transversion mutations from GC to TC [9, 10]. This process leads to cellular damage, genetic mutations, and tumor development, making it the most toxic site of AFB1. The second toxic site is the lactone ring within the coumarin nucleus, which is prone to opening in alkaline conditions to form sodium or ammonium salts, thereby reducing its toxicity. The cleavage of the lactone ring in AFB1 results in a non-fluorescent compound, with a 450-fold decrease in mutagenicity and an 18-fold reduction in toxicity [11]. The third toxic site is located on the cyclopentenone ring of AFB1, specifically at positions 1, 2, 3, and 14. The detoxification pathway for this site generally involves hydroxylation of the cyclopentenone ring [12].

The liver is the primary target organ for AFs, with AFB1 capable of causing hepatitis, liver cancer, and even death. It is a potent carcinogen [13, 14]. Therefore, different countries and regions have established specific limits for AFB1 in various food products. For example, the European Commission has stipulated that the maximum allowable level of AFB1 in any product intended for direct human consumption is 2 μg/kg. Due to the high toxicity and widespread occurrence of AFB1, the prevention and degradation of AFB1 in food are essential. Current preventive strategies mainly involve the application of pesticides and antimicrobial agents before grain harvest, and post-harvest drying, storage, vacuum packaging, preservatives, and pesticide use to reduce the contamination of grains by AFs. However, these preventive measures cannot completely eliminate aflatoxin contamination. Consequently, research on post-harvest degradation of AFs has been widely conducted by scholars, employing physical, chemical, and biological methods in an attempt to degrade and transform AFB1.

Physical degradation measures for AFs mainly include high temperature [15], irradiation [16], and adsorption [17]. Chemical degradation measures include organic acid acidification [18], ammoniation [19], and ozone treatment [20]. These methods are somewhat effective. However, their application is limited in actual production due to high costs, loss of nutritional components in the product after treatment, and decline in sensory quality. The safety and effectiveness of these physical and chemical methods need further consideration [21]. In contrast, microbial degradation of AFs is highly efficient, safe, and environmentally friendly. It can reduce the content of AFs in food and its bioavailability in living organisms.

This review mainly focuses on the degradation of AFB1 mediated by microorganisms or enzymatic actions. It discusses the microorganisms and enzymes involved in AFB1 degradation, their characteristics, and the toxicity of the degradation products. In addition, it explores the differences in the active components of aflatoxin-degrading bacteria and fungi. Finally, based on the structural identification of the degradation products, a possible enzymatic degradation mechanism for AFB1 is proposed.

Microbial degradation is a green and efficient biotechnology that utilizes the enzymatic systems of various microorganisms to transform AFB1 into other derivatives, providing new ideas for eliminating the hazards of AFB1. In recent years, significant progress has been made in the research on microbial degradation of AFB1, with multiple microorganisms reported to have the ability to degrade AFB1. However, the degradation efficiency, action sites, and mechanisms of microorganisms on AFB1 vary due to differences in strains. The most extensively studied microorganisms currently include bacteria such as *Bacillus*, *Actinomycetes* (*Mycobacterium*, *Rhodococcus*, and *Streptomyces*), and *γ-Proteobacteria*, as well as fungi, such as *Aspergillus* and *Basidiomycota*, which possess strong capabilities for AFB1 degradation.

## AFB1 Degradation by Bacteria

### AFB1 Degradation by *Bacillus*

*Bacillus velezensis* [22], *Bacillus licheniformis* [23], *Bacillus subtilis* [11, 24, 25], and *Bacillus shackletonii* [26], *Bacillus cereus* and *Bacillus mojavensis* [11], all possess the ability to degrade AFB1. However, the degradation products and mechanisms are not yet fully understood. The *aiiA* gene, which encodes N-acyl homoserine lactonase (AHL), has been identified in some AFB1-degrading strains such as *B. subtilis*, *B. cereus*, and *B. mojavensis* [11]. The presence of lactonase may act on the lactone ring of AFB1, reducing its toxicity by breaking the ring. Kumar *et al*. [27] found that the cell-free supernatant of *Bacillus albus* YUN5, isolated from Korean fermented soybean paste (doenjang), could degrade 76% and 99% of AFB1 and aflatoxin G1 (AFG1), respectively. Analysis of the degradation products suggested that the dibenzofuran ring or lactone ring are the primary sites of action.

The degradation of AFB1 by *Bacillus* is mostly enzyme-mediated, and the active ingredients for AFB1 degradation are mostly cell-free supernatants, indicating that extracellular enzymes are at play. However, there are also cases where intracellular enzymes play a major role. It is speculated that the main site of action is the lactone ring. The optimal conditions for *Bacillus* to degrade AFB1 are distributed in a wide range of 35–80°C and pH 4–11. The thermal stability of the effective components is impressive, with some being able to withstand high-temperature treatments of 70–80°C, and even 100°C, without losing activity. Some degradation products, after being tested for toxicity using *Artemia salina* and lymphocyte cells, showed reduced toxicity. After Ames mutagenicity testing, the mutagenicity was found to have disappeared.

### AFB1 Degradation by *Actinomycetes*

AFs is a category of complex aromatic compound containing α, β-unsaturated lactone groups, which is highly chronically toxic and carcinogenic to animals, including humans. Although it is relatively difficult to biodegrade, some species of actinomycetes are known to degrade AFs. For example, *Mycobacterium smegmatis* [28] and *Mycobacterium fluoranthenivorans* [29, 30] can degrade AFB1. Additionally, F420/H2-dependent reductases have been identified in mycobacterial cultures, which can utilize the deazaflavin cofactor F420/H2 to catalyze the reduction of the α, β-unsaturated ester groups in AFs, thereby activating spontaneous hydrolysis and detoxification of the molecules [28].

Krifaton *et al*. [31] discovered that 14 species of *Rhodococcus*, including *R. erythropolis*, *R. rhodochrous*, *R. globerulus*, and *R. pyridinivorans*, are capable of degrading over 80% of AFB1 within 72 h and can successfully eliminate the genotoxicity of AFB1. Kong *et al*. [32] optimized the degradation conditions of AFB1 by *R. erythropolis* using response surface methodology, increasing the degradation rate from 28.7% to 95.8%. Eshelli *et al*. [33] further revealed that the degradation of AFB1 by *R. erythropolis* involves the cleavage of the lactone group. The degradation of AFB1 is catalyzed by enzymes produced by *R. erythropolis*, forming a β-keto acid structure, followed by the hydrolysis of the lactone ring. Subsequently, the decarboxylation of the opened lactone occurs, yielding AFD1 and AFD2 (Fig. 1). While the dibenzofuran moiety is retained, the unique lactone carbonyl and cyclopentenone ring of the AFB1 molecule are eliminated.

Several species of *Streptomyces*, including *S. cacaoi* subsp. *asoensis* [34], *S. lividans* and *S. aureofaciens* [33], have also been found to possess the ability to degrade AFB1. The lactone group in the degradation products has been observed to undergo cleavage. However, the specific degradation mechanisms remain unclear.

The active components of AFB1 degradation by strains of *Mycobacteria*, *Rhodococcus*, and *Streptomyces* within *Actinomycetes* are distributed both intracellularly and extracellularly. The main target sites of these active components are the α, β-unsaturated lactone groups, involving hydrolysis of the lactone bond and reduction of the α, β-unsaturated double bonds. Some of the degradation products, tested by Aliivibrio fischeri and SOS Chromo test, showed a decrease in toxicity.

### AFB1 Degradation by γ-proteobacteria

**Fig. 2:**
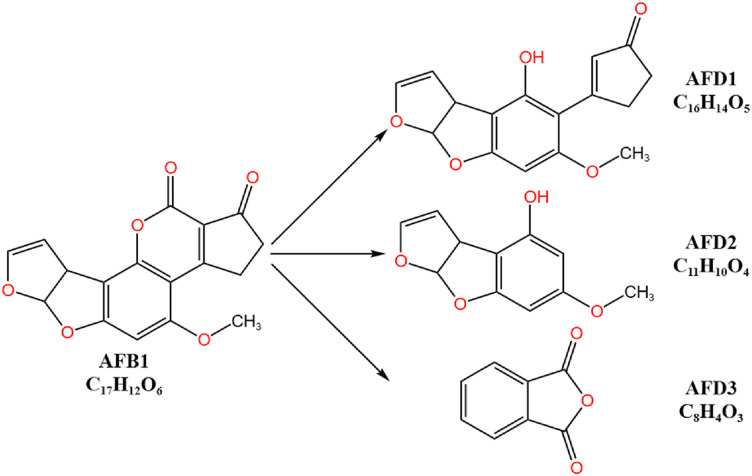
*Pseudomonas putida* biotransforms AFB1 into structurally different metabolites, including AFD1, AFD2, and AFD3 [36]. *P. putida* biotransforms AFB1 into structurally different metabolites: AFD1, AFD2, and AFD3. The lower toxicity of these metabolites is attributed to modifications in the furan ring and coumarin lactone ring. The proposed mechanism involves opening of the AFB1 lactone ring, followed by decarbonylation to form AFD1 and AFD2. The formation mechanism of AFD3 remains unclear.

Within the *γ-proteobacteria*, *Pseudomonas aeruginosa* [35], *Pseudomonas putida* [36], *Stenotrophomonas maltophilia* [37], and *Escherichia coli* [38] possess the ability to degrade AFB1, and the degradation is mediated by enzymatic processes. Among them, *E. coli* CG1061 can degrade AFB1 into C_16_H_14_O_5_ and other metabolites. The reduction in toxicity of the degradation products has been confirmed through *in vitro* experiments using chicken hepatoma cells (LMH) and *in vivo* experiments in mice [38]. *P. putida* can biotransform AFB1 into structurally different metabolites, including aflatoxin D1 (AFD1), aflatoxin D2 (AFD2), and phthalic anhydride (AFD3) (Fig. 2). These metabolites have lower toxicity due to modifications in the furan ring and coumarin lactone ring [36]. It is hypothesized that in the presence of *P. putida*, the lactone ring of AFB1 is opened, followed by decarbonylation reactions to produce AFD1 and AFD2. The mechanism of AFD3 formation, however, remains unclear.

The active components of AFB1 degradation by *γ-proteobacteria* are mostly distributed in the cell-free supernatant and are all enzyme-mediated. Protease K can significantly inhibit their activity. However, the target sites of the active components are not clear and require further research and analysis.

### AFB1 Degradation by Other Bacteria

In addition, bacteria such as *Flavobacterium aurantiacum* [39], *Myxococcus fulvus* [40], and *Tetragenococcus halophilus* [41] have also been found to possess the ability to degrade AFB1. Among them, Zhao *et al*. [40] discovered that the supernatant of *M. fulvus* ANSM068 strain cultured for 48 h achieved a degradation rate of 71.89% for AFB1. They further isolated the *Myxococcus* Aflatoxin Degrading Enzyme (MADE). When treated with 100 U/ml MADE, AFG1 and aflatoxin M1 (AFM1) were significantly degraded by 96.96% and 95.80%, respectively.

In summary, certain strains of bacteria such as *Bacillus*, *Actinomycetes*, and *γ-Proteobacteria* possess the ability to degrade AFB1 through microbial degradation. The degradation process is mostly enzyme-catalyzed, with the active components originating from intracellular or extracellular enzymes of the microorganisms. The degradation products generally exhibit reduced toxicity compared to AFB1. However, further breakthroughs are needed in the research of the action sites, characteristics of active components, and degradation mechanisms of AFB1 by bacterial strains. For example, the thermal stability mechanism of the active components in *Bacillus*, the degradation pathways of AFB1 by *Actinomycetes*, and the main action sites and degradation mechanisms of AFB1 by *γ-Proteobacteria* are still under investigation.

## AFB1 Degradation by Fungi

### AFB1 Degradation by *Aspergillus*

**Fig. 3:**
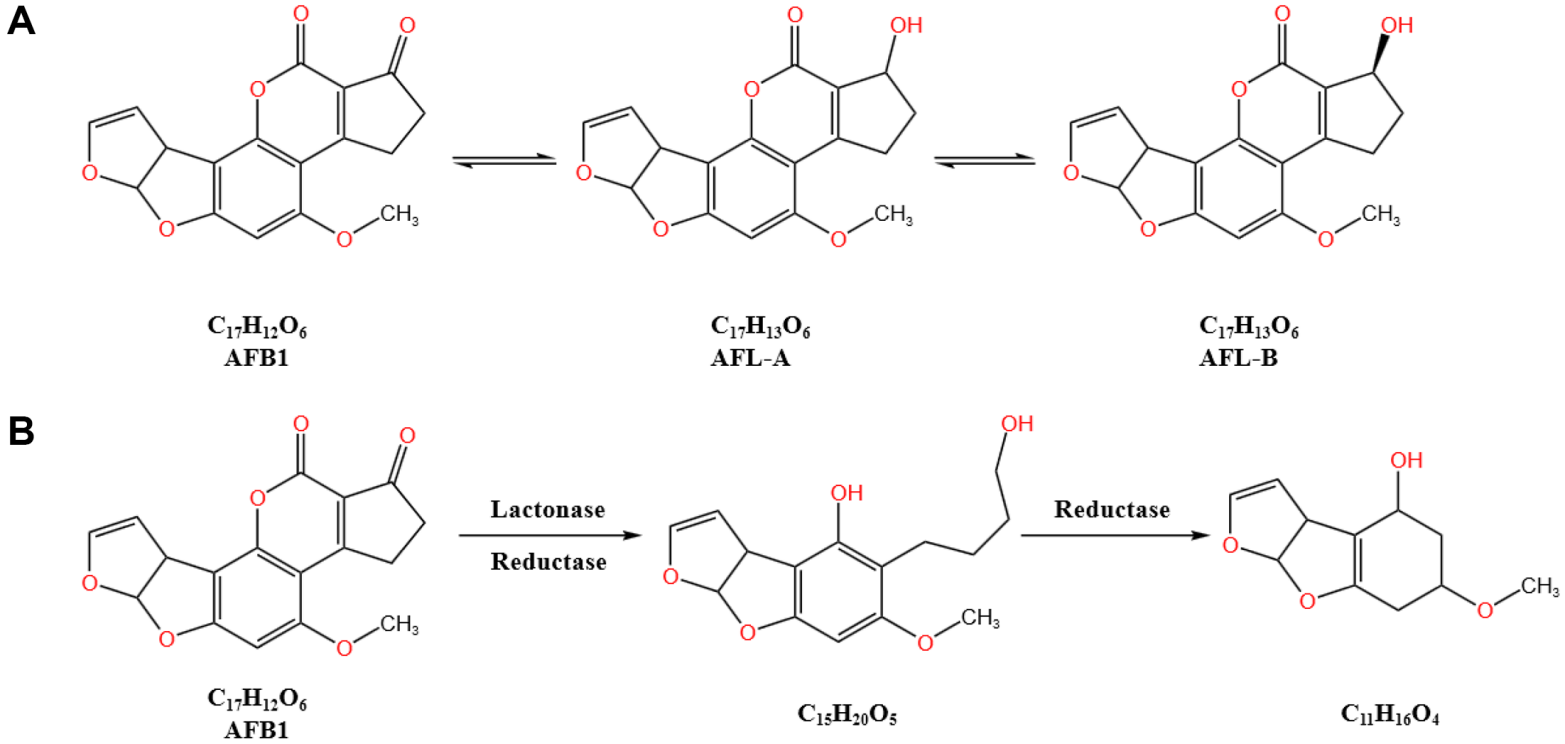
Proposed aflatoxin B1 degradation pathways. (**A**) AFB1 is converted into aflatoxinol (AFL) under the action of non-toxigenic *Aspergillus flavus* [42]; (**B**) The degradation products of AFB1 transformed by lactonase and reductase secreted by non-toxigenic *Aspergillus flavus* [44]. Non-toxigenic strains of *A. flavus* can reduce the cyclopentenone carbonyl group in AFB1, converting it into aflatoxinol-A (AFL-A) [42]. AFL-A can be further transformed into aflatoxinol-B (AFL-B) under acidic pH conditions.These strains can also convert aflatoxinol (AFL) back to AFB1, mediated by intracellular enzymes of *A. flavus*. Xing *et al*. [44] isolated two non-toxigenic strains of *A. flavus* JZ2 and GZ15 from peanuts. After co-incubation for 9 days, two new metabolites, C_15_H_20_O_5_ and C_11_H_16_O_4_, were identified. The key toxic lactone and furan rings were disrupted and hydrogenated, suggesting that lactonase and reductase enzymes may be involved in the degradation process.

Although AFB1 is generally produced by *A. flavus* within the genus *Aspergillus*, Nakazato *et al*. [42] found that non-toxigenic strains of *A. flavus* can reduce the cyclopentenone carbonyl group in AFB1 to convert it into aflatoxinol-A (AFL-A). Subsequently, AFL-A can be transformed into aflatoxinol-B (AFL-B) under acidic pH conditions. Moreover, these strains can also convert aflatoxinol (AFL) back to AFB1, a process mediated by intracellular enzymes of *A. flavus*, as shown in Fig. 3A. Over time, the total amounts of AFL and AFB1 gradually decrease and can be further metabolized by the fungus into unknown substances [43].

Xing *et al*. [44] further isolated two non-toxigenic strains of *A. flavus*, JZ2 and GZ15, from peanuts, which were capable of degrading AFB1. After co-incubation for 9 days, the degradation rates reached 53.7% and 80.9%, respectively. Two new metabolites, C_15_H_20_O_5_ and C_11_H_16_O_4_, were identified. The key toxic lactone and furan rings were disrupted and hydrogenated, suggesting that lactonase and reductase enzymes may be involved in the degradation process, as shown in Fig. 3B. Additionally, Fang *et al*. [45] found that the strain *A. niger* RAF106 possesses intracellular enzymes or proteins with excellent thermostability that can degrade AFB1 into non-toxic metabolites. Zhang *et al*. [46] reported that the degradation of AFB1 by *A. niger* ND-1 is an enzyme-mediated process occurring extracellularly. However, the exact mechanisms and products of AFB1 degradation by *A. niger* remain unclear in the aforementioned studies.

The active components of non-toxigenic *Aspergillus* fungi that degrade AFB1 are mostly distributed in the intracellular space or in the cell-free culture supernatant, and all of them function through enzymatic action. However, the nature of these active components and their specific sites of action are not yet clear and require further research and analysis.

### AFB1 Degradation by *Basidiomycota*

It has been found that several *Basidiomycota*, including *Pleurotus ostreatus* [47, 48], *Panus neostrigosus* [49], *Lentinus strigosus* [50] and *Armillariella tabescens* [51-53] possess the ability to degrade AFB1.

Motomura *et al*. [47] discovered that an extract from *P. ostreatus* contains an extracellular enzyme with a molecular weight of 90 kDa. This enzyme exhibits optimal activity at 25°C and pH 4-5 and is capable of cleaving the lactone ring of AFB1. Söylemez *et al*. [49] found that the culture filtrate of *P. neostrigosus* contains an aflatoxin-degrading enzyme with a molecular weight of 38 kDa. Co-incubation for 1 h can achieve a 49% degradation rate of AFB1. Subsequently, it was discovered that the culture filtrate of *L. strigosus* can degrade AFB1 by up to 67%. The toxicity of the degradation products was reduced by 20% as assessed by the lethality test using *A. salina* [50].

Liu *et al*. [51, 52] and Cao *et al*. [53] isolated a multi-enzyme system from *A. tabescens* that can degrade 90% of AFB1 toxin within 48 h. The Ames test confirmed that the metabolites lost their mutagenic toxicity. This multi-enzyme system was initially named Aflatoxin Detoxifying Enzyme (ADTZ). However, its oxidative properties indicated that it is an oxidase. After purification and separation, it was renamed aflatoxin-oxidase (AFO). This enzyme acts on the bis-furan ring of AFB1, initially converting it into an epoxide, which then undergoes hydrolysis to produce dihydrodiol. In subsequent hydrolysis steps, the bis-furan ring is opened.

The active components of *Basidiomycota* that degrade AFB1 are mostly distributed in the intracellular space or in the culture filtrate, and all of them function through enzymatic action. The molecular weights of the key enzymes isolated and purified are in the range of 38 kDa to 90 kDa, and the toxicity of the degradation products is significantly reduced.

In summary, researchers have previously identified numerous strains of bacteria, *Aspergillus* species, and *Basidiomycota* capable of degrading AFB1. Some scholars have also conducted detailed studies on the structures and toxicities of the degradation products. However, compared with the screening of degrading strains, research on the structures, degradation pathways, and mechanisms of the products is relatively limited and mostly inferred. The insufficient in-depth study of the mechanisms of microbial aflatoxin degradation and the lack of clarity regarding their safety in biological systems have also restricted their application in production. With deepening research on degrading microorganisms, the focus has shifted from the initial screening of degrading strains and the toxicity of degradation products to the in-depth study of degradation products, pathways, and mechanisms, which currently represent the challenging issues in the microbial degradation of AFB1.

## Research Progress on the Degradation Mechanisms of AFB1 by Microorganisms or Enzymatic Degradation

Although it has been proven that microorganisms can degrade AFB1 through enzymatic degradation effects, studies on the degradation mechanisms, structures, and toxicity of the degradation products are still limited. This has led to difficulties in practical applications and potential safety concerns. By purifying specific bio-enzymes, direct degradation of aflatoxin can be achieved. To date, many oxidases, peroxidases, reductases, laccases, lactonohydrolases, and other enzymes from microorganisms have been found to remove or degrade aflatoxin *in vitro* or in matrices [54]. However, the enzymatic degradation pathways of aflatoxin have not been fully elucidated, and clear sequential changes have not yet been established. This review attempts to classify the microbial enzymatic degradation of AFB1 into three mechanisms: enzymatic oxidation, enzymatic reduction, and enzymatic hydrolysis.

## Enzymatic Oxidative Degradation Mechanism

### Laccase-Mediated Oxidative Degradation

Laccase is a polyphenol oxidase widely distributed in bacteria and fungi, capable of catalyzing the degradation or polymerization of lignin. It typically contains four copper ions, which are crucial for the single-electron oxidation of reducing substrates and the enzyme-catalyzed oxidation using O_2_ as a mediator. Laccase can also catalyze the oxidation of phenolic compounds and aromatic amines, while reducing molecular oxygen to water. Due to its relatively low redox potential, it cannot oxidize substances with high redox potentials, such as non-phenolic components of lignin. However, by using redox mediators, the oxidative activity of laccase can be extended to non-phenolic substrates, thereby expanding its substrate range. After being oxidized by laccase, the mediator diffuses to the active sites of recalcitrant compounds with high redox potentials or large molecular weights and oxidizes them [55]. Given that the coumarin structure of AFB1 is a derivative of lignin monomer coniferyl alcohol, laccase can achieve the oxidative degradation of AFB1 through enzymatic catalysis.

### Fungal Laccase-Mediated Oxidative Degradation of AFB1

Branà *et al*. [56] obtained a crude extract with laccase activity from the spent mushroom substrate (SMS) of the edible fungus *Pleurotus eryngii*. Incubation at 25°C for 7 days resulted in the degradation of 90% of AFB1. The SMS crude extract is easily obtainable, environmentally friendly, non-toxic, and cost-effective. Martina *et al*. [57] purified a laccase (Lac2) from *Pleurotus pulmonarius*. In a sodium acetate buffer solution (1 mM) at pH 5 and 25°C, direct oxidative degradation of AFB1 reached 23% after 72 h of incubation. The degradation rate of AFB1 was significantly enhanced to over 70% when single redox mediators (10 mM) such as 2,2'-azino-bis (3-ethylbenzothiazoline-6-sulfonic acid) diammonium salt (ABTS), acetosyringone (AS), and syringaldehyde (SA) were added. This indicates that the degradation efficiency of Lac2 on AFB1 can be improved through redox mediator-mediated systems, *i.e.*, laccase-mediator system (LMS). Alberts *et al*. [58] found a significant correlation between laccase activity and AFB1 degradation in liquid cultures of strains such as *Peniophora* and *P. ostreatus*. They reported that purified laccase from *Trametes versicolor* achieved an AFB1 degradation rate of over 85%. The study showed that AFB1 was significantly degraded and its mutagenicity was markedly reduced when treated with purified laccase. Zhou *et al*. [59] purified and characterized a laccase (Lac2) from *Cerrena unicolor* 6884, which catalyzes the degradation of AFB1. The laccase-mediated system, in the presence of redox mediators such as 1 mM AS, SA, and ABTS, significantly enhanced the degradation activity of lac2 against AFB1.

Loi *et al*. [60] utilized *Saccharomyces cerevisiae* to recombinantly express Ery4 laccase from *Pleurotus eryngii* and found that it could degrade AFB1 by 73%, in the presence of laccase-mediator systems (LMSs). Song *et al*. [61] recombinantly expressed Lac2 from the large fungus *Pleurotus pulmonarius* using *Pichia pastoris*. The recombinant Lac2 was not sensitive to heat and could degrade 99.82% of AFB1 under the mediation of AS at pH 7 and 37°C after 1h of incubation.

**Fig. 4:**
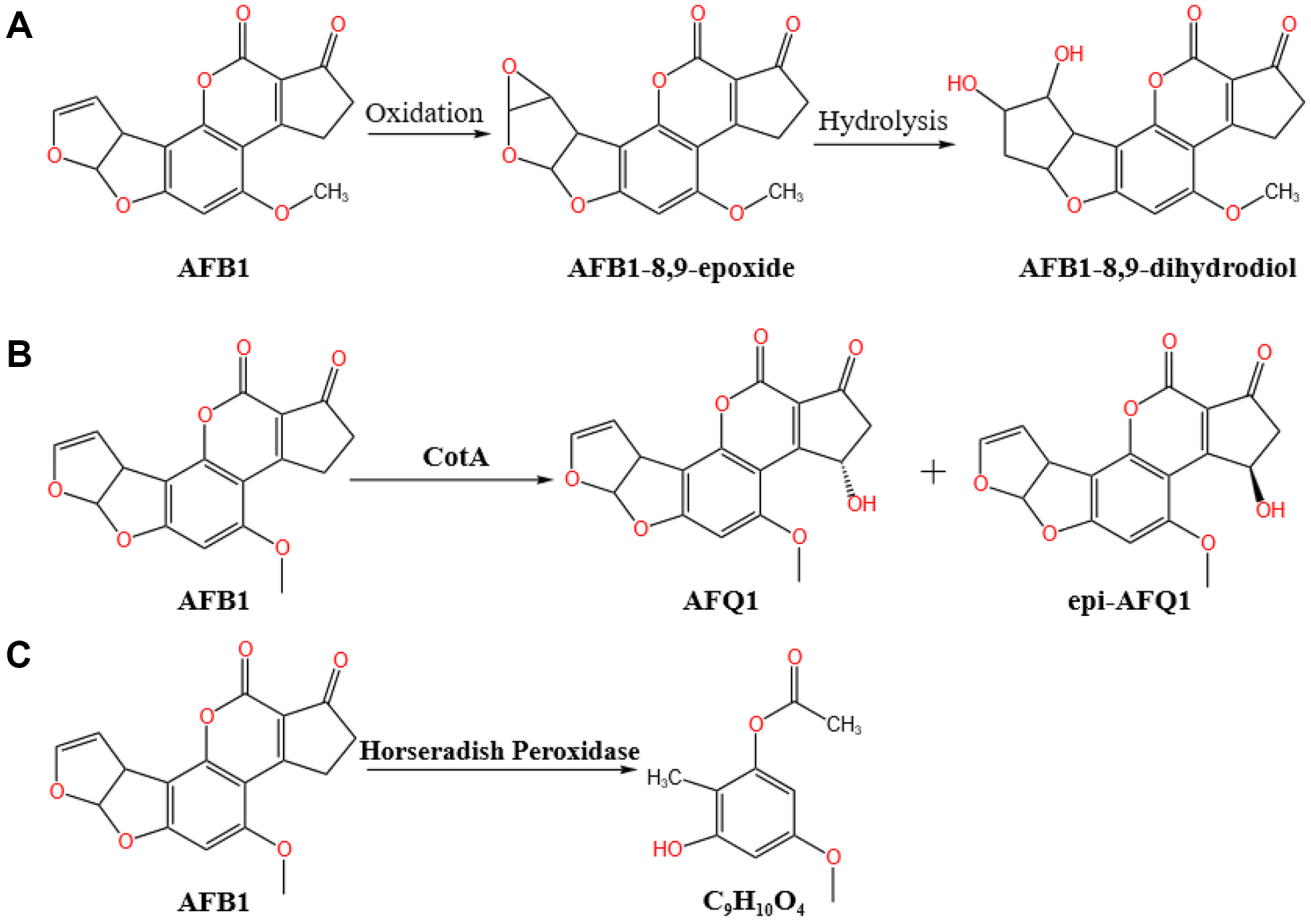
Proposed aflatoxin B1 degradation pathways. (**A**) AFB1 is converted into AFB1-8,9-dihydrodiol by laccase [63]; (**B**) AFB1 is converted into AFQ1 and epi-AFQ1 by the recombinant CotA laccase from *Bacillus licheniformis* ANSB821 expressed in *Escherichia coli* [70]; (**C**)AFB1 is converted to C_9_H_10_O_4_ by horseradish peroxidase [75]. Zaccaria *et al*. [63] conducted a comprehensive quantum mechanical analysis of laccase, revealing that the detoxification efficiency of AFB1 is influenced by enzyme-substrate affinity and environmental factors. In the presence of hydrogen atoms, AFB1 undergoes structural rearrangement, forming an epoxide (AFB1-oxide) at the terminal ring. The epoxide intermediate is further hydrolyzed to form AFB1-8,9-dihydrodiol (AFB1-diol). Guo *et al*. [70] used *E. coli* to express the recombinant rCotA from *Bacillus licheniformis* ANSB821. In addition to its laccase activity, rCotA could catalyze the direct oxidation of AFB1 to AFQ1 and epi-AFQ1. Zhou *et al*. [75] covalently immobilized horseradish peroxidase (HRP) on alginate/chitosan/montmorillonite microspheres to form SA/CS/MON-HRP adsorption/enzymatic degradation microspheres. Mass spectrometry analysis of the degradation products catalyzed by HRP revealed that the lactone ring and bis-furan ring of AFB1 were disrupted.

The enzymatic oxidation and degradation mechanism of AFB1 by fungal laccases is not yet clear, and there are significant differences in the degradation mechanisms of AFB1 among laccases from different fungal sources. Liu *et al*. [62] used *S. cerevisiae* to recombinantly express the laccase gene from *Trametes* sp. C30. The recombinant laccase could effectively degrade AFB1, with a degradation rate as high as 91%. The degradation products included C_16_H_22_O_4_, C_14_H_16_N_2_O_2_, C_7_H_12_N_6_O, and C_24_H_30_O_6_. These degradation products were tested using HepG2 cells and *in vivo* assays, and their cytotoxicity and hepatotoxicity were significantly reduced. Two possible degradation pathways were proposed: (1) AFB1 consecutively loses -CO, and then the double bond in the furan ring breaks after reacting with H_2_O, H+, and -NH_2_; (2) After losing -CO, AFB1 undergoes decarbonylation, and the double bond breaks through additional reactions with H+. The two toxic active sites in AFB1, including the double bond in the furan-furan ring and the lactone ring in the coumarin part, are disrupted. Zaccaria *et al*. [63] conducted a comprehensive quantum mechanical analysis of laccase and found that the detoxification efficiency of AFB1 by laccase is affected by the enzyme-substrate affinity and the environment. Laccase does not directly interact with the lactone ring of the toxin to achieve degradation. When hydrogen atoms are present in the environment, AFB1 undergoes structural rearrangement, forming an epoxide AFB1-oxide at the terminal ring, which is further hydrolyzed to AFB1-8,9-dihydrodiol (AFB1-diol), as shown in Fig. 4A.

### Bacterial Laccase-Mediated Oxidative Degradation of AFB1

Cai *et al*. [64] isolated a strain of *Sphingomonas acidoaminiphila* CW117 capable of degrading AFB1. The study found that the active degrading component was present in the cell-free supernatant, and the activity remained stable even at 90°C. The recombinant laccase rLC1 obtained by expression in *E. coli* could degrade 29.3% of AFB1 within 24 h. However, under the same conditions, the cell-free supernatant could degrade 76.7% of the toxin, indicating that CW117 degrades AFB1 through the combined action of laccase and trace oxidants. Xiong *et al*.[65] isolated a strain of *Bacillus amyloliquefaciens* B10 that could degrade 2.5 mg/ml of AFB1 within 96 h. The active degrading component was purified as a novel laccase. When the laccase gene was recombinantly expressed in *E. coli*, the optimal degradation conditions for the novel laccase were found to be 40°C, pH 6-8. Magnesium ions (Mg^2+^) and dimethyl sulfoxide (DMSO) could enhance the degradation activity of the B10 laccase. Bian *et al*. [66] used *E. coli* to recombinantly express the laccase gene from *Bacillus vallismortis* fmb-103. The recombinant laccase fmb-rL103 could degrade AFB1 without a mediator, with a degradation rate of 60% at pH 7.0 and 37°C.

The CotA protein, a bacterial laccase from the genus *Bacillus*, which is a component of the endospore coat, has high non-specific oxidizing ability and has been proven to oxidize polycyclic aromatic hydrocarbons [67]. Wang and Bai *et al*. [68] used *E. coli* to recombinantly express the CotA gene (1,542 bp) from *B. subtilis*, encoding a laccase composed of 513 amino acids. The recombinant laccase BsCotA could degrade 98% of AFB1 in the presence of syringic acid as a mediator, and the degradation products were confirmed to be detoxified. Sun *et al*.[69] cloned and expressed the CotA laccase from *B. licheniformis* ZOM-1 in *E. coli*, showing that this laccase could degrade AFB1. The optimal catalytic conditions were 80°C and pH 9.0, and the oxidative degradation products had significantly reduced toxicity. Guo *et al*. [70] used *E. coli* to express the recombinant rCotA from *B. licheniformis* ANSB821. In addition to its laccase activity, rCotA could catalyze the direct oxidation of AFB1 to AFQ1 and epi-AFQ1, as shown in Fig. 4B. The cytotoxicity of the degradation products was significantly reduced when tested with human hepatocytes (L-02).

*Basidiomycota* and bacteria can both produce laccase to achieve enzymatic oxidative degradation of AFB1, with or without the participation of mediators. Comparisons show that bacterial laccases exhibit superior properties to fungal laccases, such as higher thermal stability, broader substrate specificity, and wider pH tolerance. These characteristics make bacterial laccases more suitable candidates for the degradation of AFs. Given the widespread distribution of laccase genes, it is also possible to heterologously express and produce laccase-encoding genes in engineered strains for aflatoxin degradation. Heterologous expression of recombinant enzymes can achieve efficient production, reducing labor and economic costs while offering scalability, high enzyme yields, and improved enzyme stability. Additionally, protein engineering can be used to optimize enzyme properties to enhance catalytic activity and substrate specificity. Overall, the application of recombinant enzymes in AFB1 degradation provides a promising and cost-effective approach with potential industrial value.

### Manganese Peroxidase (MnP)-Mediated Oxidative Degradation

MnP is a Mn^2+^-dependent peroxidase with a redox potential as high as 1.5 V, which is much higher than that of laccase (790 mV) [71]. The presence of carboxylates can further enhance the oxidative capacity of MnPs. Wang and Qin *et al*. [71] discovered eight MnPs from different lignocellulose-degrading fungi could degrade AFB1 through radical attack in the presence of oxalate. They also pointed out that fungi capable of degrading lignin can degrade organic compounds, including mycotoxins, with MnP being the primary enzyme responsible for lignin oxidation and depolymerization in these fungi. Subsequent mass spectrometry analysis revealed that MnP5 and MnP6 could oxidize the C=C double bond at positions 8 and 9 in the furan ring of AFB1 and introduce an oxygen atom into the molecule to form AFBO.

Yehia *et al*. [48] isolated a MnP with a molecular weight of 42 kDa from the culture supernatant of *Pleurotus ostreatus*, encoded by a 497-bp gene. Under conditions of 1.5 U/ml enzyme activity at pH 4-5 and 25°C, 90% of AFB1 could be degraded within 48 h. Wang *et al*. [72] isolated MnP from *Phanerochaete sordida* YK-624, which could degrade 86% of AFB1 within 48 h at an activity level of 5 nKat. The mutagenicity was significantly reduced. Analysis of the degradation products revealed that AFB1 was first oxidized to AFBO by MnP and then hydrolyzed to AFB1-diol, effectively eliminating its mutagenic activity. Xia *et al*. [73] used *Kluyveromyces lactis* GG799 to recombinantly express the MnP-encoding gene from *Phanerochaete chrysosporium*. The fermentation supernatant of the recombinant strain achieved a 75.71% degradation rate of AFB1, with the degradation product identified as AFB1-diol.

In summary, MnPs from different sources exhibit varying efficiencies in AFB1 degradation. The typical degradation products of AFB1 by MnP are AFB1-diol or AFBO.

### Catalase-Mediated Oxidative Degradation

Xu *et al*. [74] cloned the catalase gene KatA from *P. aeruginosa* into *E. coli*. The recombinant catalase had a molecular weight of approximately 55.6 kDa. In the presence of eugenol, the recombinant catalase achieved a degradation rate of 38.79% for AFB1. However, the degradation mechanism of AFB1 by catalase and the toxicity of the degradation products are still unclear.

### Peroxidase-Mediated Oxidative Degradation

Zhou *et al*. [75] found that when horseradish peroxidase (HRP) was covalently immobilized on alginate/chitosan/montmorillonite microspheres to form SA/CS/MON-HRP adsorption/enzymatic degradation microspheres, the degradation efficiency of AFB1 by HRP was increased by 17.25% in the presence of 0.1 mmol concentration of H_2_O_2_. They also performed mass spectrometry analysis on the degradation products of AFB1 catalyzed by HRP and speculated that the degradation products are as shown in Fig. 4C, indicating that the lactone ring and bis-furan ring of AFB1 were disrupted.

Sibaja *et al*. [76] used commercial peroxidase (POD) at a concentration of 0.015 U/ml in 100 mmol/l phosphate buffer at pH 7-8 to incubate AFB1 at 30-40°C for 8 h, achieving 97% degradation of AFB1 at a concentration of 0.5 μg/l. Additionally, the addition of POD to milk and beer could degrade AFB1, providing a novel approach for the degradation of AFs. Qin *et al*. [77] used recombinant *E. coli* to express the dye-decolorizing peroxidase gene BsDyP from *B. subtilis* SCK6. The purified peroxidase BsDyP could oxidize multiple substrates and effectively degrade various mycotoxins, including AFB1, zearalenone (ZEN), and deoxynivalenol (DON), in the presence of Mn^2+^. The degradation products were identified as AFB1-diol, 15-hydroxy-ZEN, and C_15_H_18_O_8_, all of which showed significantly reduced toxicity. Loi *et al*. [78] used recombinant *E. coli* to express the N246A variant of DypB from *Rhodococcus jostii*, a type B dye-decolorizing peroxidase (Rh-DypB). They found that Rh-DypB could enzymatically degrade AFB1 *in vitro*. After incubation at 25°C for 4 days, AFB1 was quantitatively converted to AFQ1, a compound with significantly reduced toxicity.

### Uncharacterized Oxidase-Mediated Oxidative Degradation

Liu *et al*. [51, 52] and Cao *et al*. [53] isolated a multienzyme complex from *A. tabescens*, which could degrade 90% of AFB1 toxin within 48 h. The Ames test confirmed that the metabolites had lost their mutagenic toxicity. The enzyme acts on the bis-furan ring of AFB1, initially converting AFB1 into an epoxide, which is then hydrolyzed to produce dihydrodiol. In subsequent hydrolysis steps, the bis-furan ring is opened. Based on its characteristics, the enzyme was classified as an oxidase and renamed aflatoxin-oxidase (AFO).

In summary, it can be observed that oxidases from different sources exhibit varying efficiencies in degrading AFB1. The action sites of oxidases are more concentrated on the C8, C9 double bond and the cyclopentenone group of the bis-furan ring of AFB1. The degradation products of AFB1 are generally AFB1-diol or AFQ1.

## Enzymatic Reductive Degradation Mechanism

**Fig. 5:**
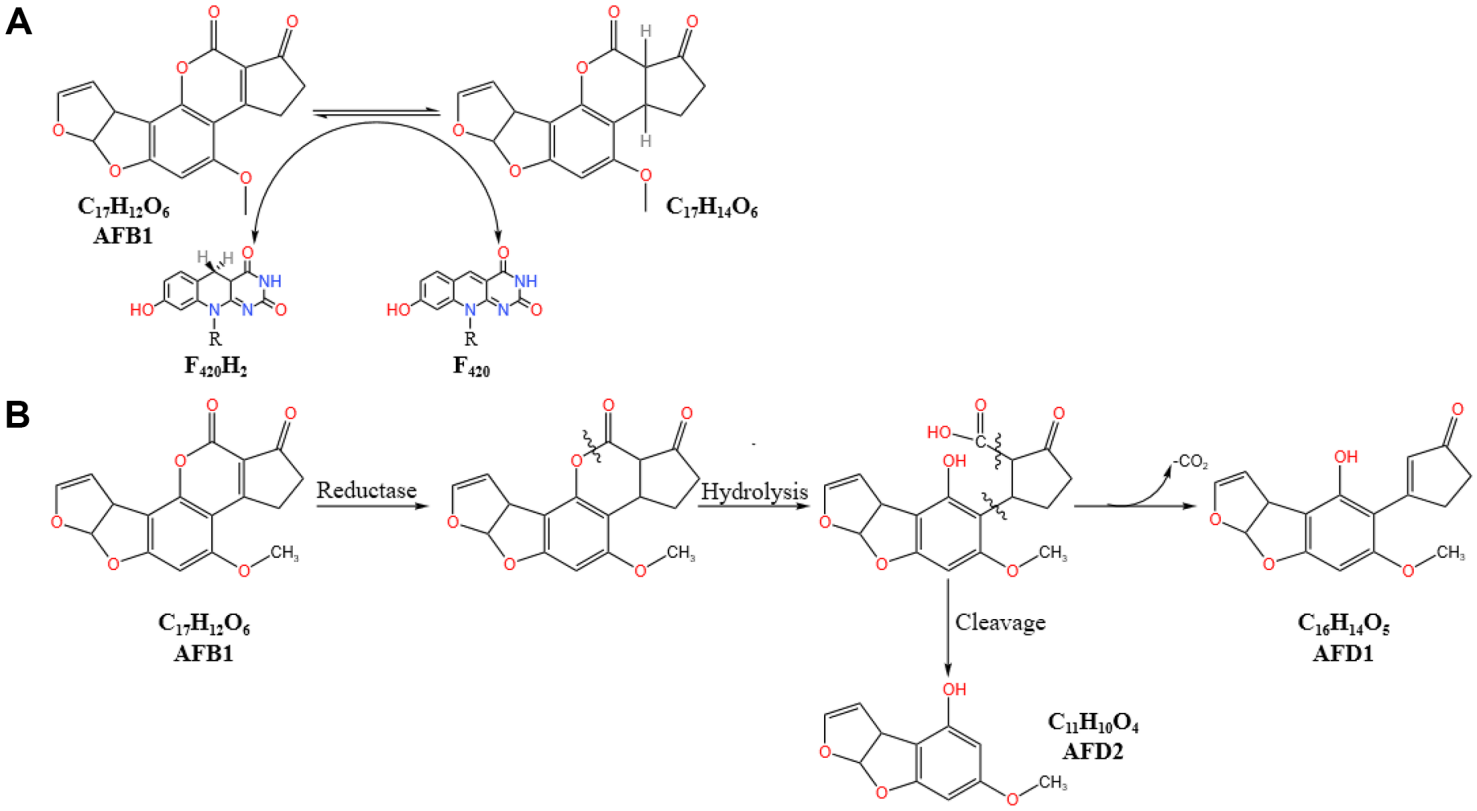
Proposed aflatoxin B1 degradation pathways. (**A**) F420H2-dependent reductase catalyzes the reduction of the α, β-unsaturated ester group in AFB1 [28]. (**B**) The enzymatic reductive degradation of AFB1 by the lysin biosynthesis redox enzyme encoded by the bacC gene in *Bacillus subtilis* UTB1 [82]. Taylor *et al*. discovered the F420H2-dependent reductase families FDR-A and FDR-B in the culture of *Mycobacterium smegmatis*.These enzymes utilize the deazaflavin coenzyme F420H2 to transfer two electrons, catalyzing the reduction of the α, β-unsaturated ester group double bond in AFB1. The reaction product is unstable and subsequently undergoes spontaneous hydrolysis and decarboxylation. Afsharmanesh *et al*. [82] discovered that the enzyme encoded by the bacC gene, a lysin biosynthesis redox enzyme, catalyzes the reduction of the double bond in the lactone ring of AFB1. Subsequent hydrolysis of the ester bond produces a carboxylic acid. The presence of α- β unsaturated bonds in the product increases its chemical reactivity, leading to a series of degradation reactions. Specifically, AFD1 is produced after decarboxylation, or AFD2 is formed after hydrolysis, with the cyclopentenone ring being cleaved.

The molecular structure of AFB1 is closely related to its toxicity. The furanone ring and the α, β-unsaturated ester structure between the lactone and furanone rings can serve as targets for reductases, thereby enabling the biodegradation of AFB1. F_420_ is a natural deazaflavin derivative and a coenzyme with a low reduction potential, participating only in the transfer of hydrides [79]. Taylor *et al*. discovered the F420-H2-dependent reductase families FDR-A and FDR-B in the culture of *M. smegmatis*. These enzymes can utilize the deazaflavin coenzyme F420-H2 to transfer two electrons to catalyze the reduction of the α, β-unsaturated ester group double bond in aflatoxin B1, as shown in Fig. 5A. The reaction product is unstable and subsequently undergoes spontaneous hydrolysis and decarboxylation [28]. Lapalikar *et al*. [80] identified and characterized the F420-H2-dependent deazaflavin reductase FDR-A from *R. erythropolis* PR4, which can reduce the α, β-unsaturated ester group of AFB1, activate spontaneous hydrolysis and detoxification, and degrade AFB1 into smaller compounds that eventually enter the citric acid cycle for metabolism. However, the stereochemistry of the reduction products and the further degradation products remain to be determined [81].

Farzaneh *et al*. [25] isolated a strain of *B. subtilis* UTBSP1 from pistachios, which could enzymatically degrade AFB1. Afsharmanesh *et al*. [82] further discovered that the enzyme encoded by the bacC gene in this strain, a lysin biosynthesis redox enzyme, catalyzes the reduction of the double bond in the lactone ring of AFB1. Subsequent hydrolysis of the ester bond produces a carboxylic acid. The presence of α-β unsaturated bonds in the product increases its chemical reactivity, leading to a series of degradation reactions. Specifically, AFD1 is produced after decarboxylation, or AFD2 is formed after hydrolysis, with the cyclopentenone ring being cleaved (Fig. 5B). F420/H2-dependent reductases preferentially reduce the double bond in the lactone ring.

## Enzymatic Lactone Ring Hydrolysis Mechanism

**Fig. 6:**
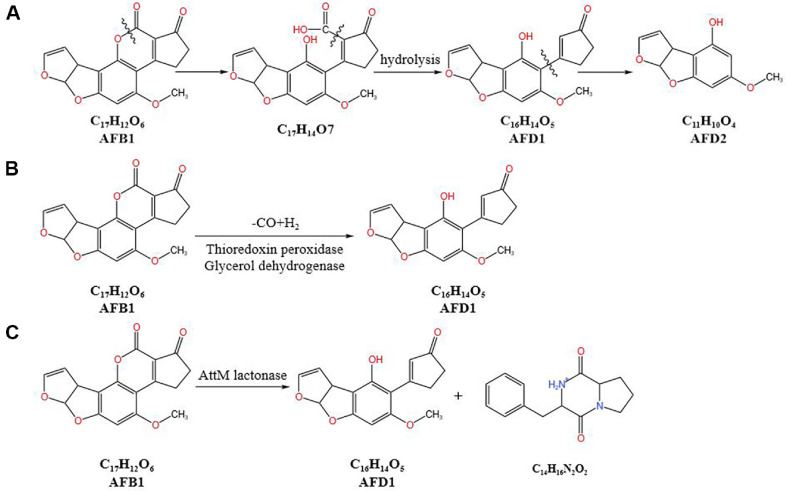
Proposed aflatoxin B1 degradation pathways. (**A**) The hydrolysis of the lactone ring of AFB1 by lactonase. [83]; (**B**) The hydrolysis of the lactone ring of AFB1 by thioredoxin peroxidase (Tpx) and glycerol dehydrogenase (GldA) [84]; (**C**) The hydrolysis of the lactone ring of AFB1 by AttM lactonase [85]. Ludmila *et al*. [83] used high-resolution mass spectrometry (FTMS) to observe that cleavage of the lactone ring forms a β-keto acid structure. Decarboxylation of the opened lactone ring produces AFD1. Alternatively, after hydrolysis, the cyclopentenone ring is cleaved to form AFD2. Shi *et al*. [84] isolated a strain of *Bacillus amyloliquefaciens* ZG08 from kimchi, which degrades AFB1 through an enzymatic process. Two potential key genes, thioredoxin peroxidase (Tpx) and glycerol dehydrogenase (GldA), hydrolyze the lactone ring of AFB1. The degradation product identified is AFD1. Cheng *et al*. [85] discovered that the AttM gene in *B. megaterium* HNGD-A6 encodes an N-acyl homoserine lactonase. The enzyme reduces the toxicity of AFB1 by disrupting its lactone structure. The detoxification process yields AFD1 and C_14_H_16_N_2_O_2_, both of which exhibit significantly reduced toxicity.

The coumarin lactone ring in AFB1 is a crucial site responsible for its toxicity. The cleavage of the lactone ring in AFB1 produces a non-fluorescent compound, reducing its mutagenicity by 450-fold and toxicity by 18-fold [11]. Microorganisms can reduce the toxicity of AFB1 by enzymatically hydrolyzing the lactone ring of the coumarin core using lactonases. For example, the mechanism by which *Rhodococcus erythropolis* ATCC 4277 degrades AFB1 is speculated to involve enzymatic hydrolysis of the lactone group [33]. A 90 kDa extracellular enzyme has been identified in the edible fungus *Agaricus bisporus*, which can enzymatically hydrolyze the lactone ring of aflatoxin. Gonzalez *et al*. [11] discovered N-acyl homoserine lactonase (AHL) encoded by the *aiiA* gene in AFB1-degrading strains of *B. subtilis*, *B. cereus*, and *B. mohavensis*. It is hypothesized that AHL lactonase can cleave the lactone ring of AFB1, thereby reducing its toxicity. Ludmila *et al*. [83] used high-resolution mass spectrometry (FTMS) to observe that lactone ring cleavage forms a β-keto acid structure, followed by decarboxylation of the opened lactone ring to produce AFD1. Alternatively, after hydrolysis, the cyclopentenone ring is cleaved to form AFD2, as shown in Fig. 6A.

Shi *et al*. [84] isolated a strain of *Bacillus amyloliquefaciens* ZG08 from kimchi, which could degrade 80.93% of AFB1 within 72 h at 37°C and pH 7.0 through an enzymatic degradation process. They over-expressed two potential key genes, thioredoxin peroxidase (Tpx) and glycerol dehydrogenase (GldA), in *E. coli* BL21, achieving AFB1 degradation rates of 60.05% and 47.16%, respectively. The lactone ring of AFB1 was hydrolyzed, and the degradation product was identified as AFD1 (Fig. 6B). Validation using HepG2 cells demonstrated a significant reduction in cytotoxicity. Cheng *et al*. [85] discovered that the extracellular enzymes of *Bacillus megaterium* HNGD-A6 could degrade AFB1, with the degradation products being AFD1 and C_14_H_16_N_2_O_2_ (Fig. 6C), both of which exhibited significantly reduced toxicity. They successfully expressed the AttM gene, which encodes an N-acyl homoserine lactonase, in *E. coli* BL21. The AttM lactonase demonstrated remarkable heat resistance, degrading 86.78% of AFB1 at pH 8.5 and 80°C. The detoxification mechanism of AttM is speculated to involve the disruption of the lactone structure of AFB1, thereby reducing its toxicity. In summary, it can be observed that hydrolytic enzymes from different sources exhibit varying efficiencies in degrading AFB1. The hydrolysis of the lactone ring makes AFD1 one of the common degradation products of AFB1.

## Discussion

Based on existing research, this review provides an overview of the microbial degradation technologies for AFB1 and their enzymatic degradation mechanisms. Microbial degradation relies on the reaction between biocatalytic enzymes and AFB1 to disrupt its structure, thereby achieving detoxification. To further illustrate the characteristics of different microorganisms or biocatalytic enzymes in terms of degradation products and mechanisms, we analyzed the molecular structures of reported degradation products. The diversity of degradation products indicates multiple transformation pathways for the biological degradation of AFB1. Most degradation products formed during enzymatic reduction and hydrolysis are due to the disruption or modification of the lactone ring in the AFB1 structure, while enzymatic oxidation targets the destruction of the furan ring or cyclopentenone structure. These ring structures are the primary toxic sites of AFs, and their disruption leads to reduced or eliminated toxicity.

Microbial or enzymatic degradation is considered the most promising method for AFB1 degradation due to its high efficiency and specificity. It offers significant advantages in preserving food nutrients and protecting the environment. However, microbial or enzymatic degradation technologies also face challenges related to degradation efficiency and culture conditions, as well as the need to control microbial growth in aflatoxin-contaminated media. Additionally, when applying biotransformation technologies for the degradation of mycotoxins in food, safety must be a priority. Strains/enzymes that have undergone safety assessments should be selected to meet the required conditions.

Enzyme engineering technologies, such as genetic recombination, directed mutagenesis, and heterologous expression, have become effective tools for improving the efficiency and operational stability of aflatoxin-degrading enzymes. From an economic perspective, this review advocates the use of aflatoxin-degrading microorganisms in animal husbandry and the food industry. However, this technology also faces challenges related to time efficiency and culture conditions, as well as the need to control the impact of microbial growth on substrate quality. Moreover, due to the high specificity and selectivity of enzymes, the use of aflatoxin-degrading enzymes can be expected to have negligible effects on food sensory quality. However, improving the yield, activity, and stability of key enzymes requires breakthroughs in both economic and technical aspects to avoid additional production costs.

## Conclusions and Prospects

Microbial or enzymatic degradation is an effective and mild detoxification measure for AFs, and significant research progress has been made to date. However, there are still deficiencies in the analysis of degradation products, structural identification, degradation mechanisms, and toxicity studies of degradation products. Therefore, this study believes that future research on microbial or enzymatic degradation of AFB1 should focus on the following aspects:

Structural Identification and Toxicity Analysis of AFB1 Degradation Products: Compared to the screening of degrading strains, research on the structure and toxicity evaluation of degradation products is relatively limited, restricting the potential application of microbial degradation measures in production. In-depth studies should be conducted on the mechanisms of microbial or enzymatic degradation of AFB1 and the toxicological activity testing of degradation products.

Economic Extraction and Application of Key Active Components for AFB1 Microbial or Enzymatic Degradation: Systematic research should be carried out on the degradation mechanisms of AFs, focusing on the active components, key enzymes, and genes involved in degradation. Through genetic engineering technologies, research should be conducted on the economical cultivation and extraction of biocatalytic enzymes to realize the application of complete enzymatic degradation pathways in production.

Integration of AFB1 Degradation Measures with Practical Applications for Long-term Degradation or Protection: Existing microbial or enzymatic degradation measures should be closely integrated with practical applications to develop safe and reliable technologies suitable for the development of animal husbandry or the food industry. Additionally, efforts should be made to prevent recontamination of treated materials with toxins and to achieve long-term degradation or protection.

With the advancement of biotechnology, microbial or enzymatic degradation of AFB1 is expected to play an important role in the prevention and control of mycotoxin contamination.
